# Long-term nitrate administration modulates sialin gene expression in the main tissues of male Wistar rats with type 2 diabetes

**DOI:** 10.17179/excli2024-8051

**Published:** 2025-03-03

**Authors:** Sajad Jeddi, Nasibeh Yousefzadeh, Vajiheh Khorasani, Maryam Zarkesh, Khosrow Kashfi, Asghar Ghasemi

**Affiliations:** 1 Endocrine Physiology Research Center, Research Institute for Endocrine Sciences, Shahid Beheshti University of Medical Sciences, Tehran, Iran; 2 Cellular and Molecular Endocrine Research Center, Research Institute for Endocrine Sciences, Shahid Beheshti University of Medical Sciences, Tehran, Iran; 3 Department of Molecular, Cellular, and Biomedical Sciences, Sophie Davis School of Biomedical Education, City University of New York School of Medicine, NY, USA

**Keywords:** sialin, nitrite, nitrate, type 2 diabetes, nitrate transporter

## Abstract

The increased sialin gene expression in the main tissues of diabetic rats is associated with decreased nitrate and nitrite levels, suggesting a counterregulatory response for reduced nitric oxide (NO) bioavailability. In this study, we hypothesized that long-term nitrate administration (6 months) would decrease sialin gene expression in rats with type 2 diabetes (T2D). Rats were assigned to two groups (n=10): T2D and T2D+nitrate, receiving nitrate in their drinking water at a concentration of 100 mg/L over 6 months. Samples from the main tissues were collected and used to measure the gene expression of sialin, as well as nitrate and nitrite levels. Nitrate-treated T2D rats had higher nitrate levels in the soleus muscle (SM) (163 %), stomach (83 %), lung (271 %), pancreas (90 %), aorta (61 %), adrenal gland (88 %), brain (145 %), liver (95 %), and heart (87 %). Nitrite levels were also higher in SM (136 %), lung (108 %), pancreas (86 %), kidney (88 %), aorta (33 %), brain (221 %), epididymal adipose tissue (eAT) (52 %), and heart (93 %), of nitrate treated T2D rats (all P<0.05). Nitrate decreased sialin gene expression in the SM (0.21-fold, P<0.001), stomach (0.37-fold, P=0.002), liver (0.21-fold, P<0.001), and eAT (0.47-fold, P=0.016) but it increased it in the intestine (1.99-fold, P<0.001), pancreas (2.01-fold, P=0.006), and the kidney (2.45-fold, P<0.001) of diabetic rats, with no effects in the lung, aorta, adrenal gland, brain, and heart. Nitrate administration restores the compensatory increase in sialin gene expression in tissues of T2D rats. However, this compensatory mechanism is not generalizable to all tissues.

## Introduction

Type 2 diabetes (T2D) ranks as the ninth leading cause of mortality worldwide (Zheng et al., 2018[53]). Its global prevalence was 4.6 % in 2000, projected to reach 12.2 % by 2045, representing an increase from 151 to 783 mil-lion (IDF, 2021[17]). The levels of nitrite/nitrate and nitric oxide (NO) have been reported to be lower in the tissues of rats with T2D, including the liver (Shokri et al., 2021[47]; Yousefzadeh et al., 2023[52]), soleus muscle (SM) (Shokri et al., 2021[47]; Yousefzadeh et al., 2023[52]), epididymal adipose tissue (eAT) (Shokri et al., 2021[47]; Yousefzadeh et al., 2023[52]), inguinal AT (Varzandi et al., 2018[49]), kidney (Yousefzadeh et al., 2023[52]), heart (Bulhak et al., 2009[6]; Yousefzadeh et al., 2023[52]), and aorta (Bitar et al., 2005[5]; Yousefzadeh et al., 2023[52]). This data represents a generalized decreased NO bioavailability in T2D that contributes to the pathophysiology of T2D (Bahadoran et al., 2015[3]; Lundberg et al., 2018[25]). Reduced NO bioavailability in T2D is associated with impaired NO synthase (NOS) (Lin et al., 2002[23]; Muhammed et al., 2012[29]) and nitrate-nitrite-NO pathways (Bahadoran et al., 2015[3]). It has been suggested that nitrate, as a NO-boosting supplement, potentiates the nitrate-nitrite-NO pathway and provides a complementary resource for the NOS pathway in T2D (Lundberg and Weitzberg, 2009[27]; Lundberg et al., 2015[26]). 

Previous studies have demonstrated that nitrate/nitrite exerts favorable metabolic effects in T2D rodent models through multiple mechanisms, including increasing browning of white adipose tissue (WAT) (Roberts et al., 2015[46]; Varzandi et al., 2018[49]), pancreatic insulin secretion (Nyström et al., 2012[31]; Ghasemi et al., 2022[10]), glucose uptake in the skeletal muscle and AT (Jiang et al., 2014[19]; Khoo et al., 2014[20]) as well as improving insulin resistance (Ohtake et al., 2015[32]) and decreasing pancreatic oxidative stress (Ghasemi et al., 2023[11]). The phospholipid bilayer restricts the free movement of nitrate molecules; therefore, nitrate needs to be transported into cells for these anti-diabetic effects to manifest. This is done through sialin, a 2NO_3_^-^/H^+^ cotransporter (Ip et al., 2020[18]; Qin and Wang, 2022[43]). Knockdown of sialin decreases (Srihirun et al., 2020[48]), whereas its overexpression facilitates (Feng et al., 2021[9]) nitrate influx into skeletal muscle cells and submandibular gland cell lines of humans, respectively. Sialin has widespread expression, including in the salivary glands, muscles, cardiovascular system, liver, pancreas, brain, AT, and kidney (Qin et al., 2012[42]; Park et al., 2020[34]; Feng et al., 2021[9]; Yousefzadeh et al., 2023[52]), suggesting its potential role in the regulation of systemic nitrate-nitrite-NO balance (Qin et al., 2012[42]; Park et al., 2020[34]; Feng et al., 2021[9]; Yousefzadeh et al., 2023[52]). 

We recently reported that T2D rats have higher sialin gene expression in the SM, liver, stomach, eAT, and adrenal gland; this increased sialin gene expression coincided with decreased nitrite and nitrate concentrations in these tissues (Yousefzadeh et al., 2023[52]). We hypothesized that increased expression of sialin in the main tissues of T2D rats is a compensatory mechanism for counteracting the decrease in NO bioavailability. Supporting this idea, sialin expression increased by 89 % in the skeletal muscle of mice with myoglobin-deficient phenotype (Park et al., 2019[33]) and by 50 % in dietary nitrate-deficient rats (Park et al., 2021[36]); both have reduced NO bioavailability. To our knowledge, alterations in sialin gene expression following nitrate administration in T2D, if any, have not been reported. Hence, this study aimed to fill this gap and assess the effects of long-term (6-month) nitrate administration at a concentration of 100 mg/L in drinking water on sialin gene expression in the main tissues of T2D rats.

## Materials and Methods

### Ethical approval

All experiments of the current study were affirmed by the published guideline of the care and use of laboratory animals in Iran (Ahmadi-Noorbakhsh et al., 2021[1]) and reported following ARRIVE guidelines (Percie du Sert et al., 2020[37]). The ethics committee of the Research Institute for Endocrine Sciences, affiliated with the Shahid Beheshti University of Medical Sciences, confirmed and approved all experimental procedures of the current study (Ethic Code: IR.SBMU.ENDOCRINE.REC.1402.075). In the present study, rats were anesthetized with an intraperitoneal (IP) injection of xylazine (10 mg/kg) and ketamine (50 mg/kg). After confirming successful euthanasia through cervical dislocation, the rat's brain was isolated first, and then other tissues were removed from the body for further study.

### Induction of T2D

Wistar rats were obtained from the Research Institute for Endocrine Sciences of Shahid Beheshti University of Medical Sciences, Tehran, Iran. Two-month-old male Wistar rats weighing 200-210 g were kept in polypropylene cages under controlled environmental conditions, including temperature maintained at 23±2 °C and a light-dark cycle of 12 hours each. Rats had unrestricted access to a standard diet and water *ad libitum* throughout the study. To induce T2D, rats were given a high-fat diet (HFD) for 2 weeks, followed by an intraperitoneal injection of streptozotocin (STZ, 30 mg/kg). One week later, fasting blood glucose was measured, and rats were considered to be diabetic if their blood glucose levels were ≥ 150 mg/dL (Ghasemi and Jeddi, 2023[13]).

### Experimental design of the study 

**Figure 1:**
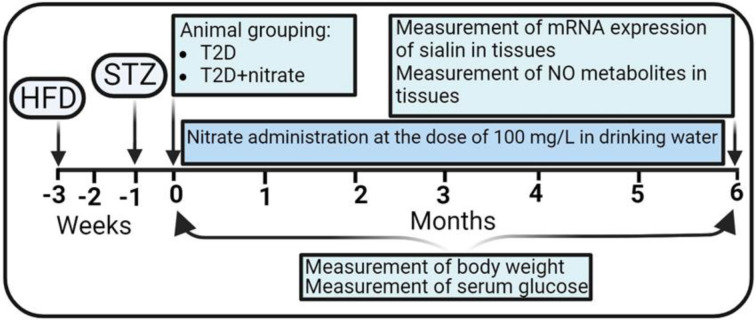
Experimental design of the study. NO, Nitric oxide; HFD, High-fat diet; T2D, Type 2 diabetes; STZ, Streptozotocin

The interventional experimental design is illustrated in Figure 1(Fig. 1). Once the T2D model was confirmed, rats at the beginning of the study (month 0) were randomly allocated into two experimental groups, T2D and T2D+nitrate (n=10 per group). The T2D+nitrate group received sodium nitrate for 6 months (100 mg/L in drinking water), whereas the T2D group received tap water only. In addition, both groups were maintained on the HFD during the study. Serum glucose and body weight were measured in all animals at the intervention's start (month 0) and end (month 6). Rats were anesthetized using ketamine/xylazine (50/10 mg/kg) at month 6, and the main tissues, including the SM, stomach, intestine, lung, pancreas, kidney, aorta, adrenal gland, brain, liver, eAT, and heart, were isolated, snap frozen in liquid nitrogen, and stored at -80 °C for further processing. Tissue sialin gene expression was measured using real-time PCR, and for all samples, NO metabolite levels (nitrate + nitrite = NOx) were determined using the Griess method. 

### Measurement of serum glucose level

Serum glucose was measured utilizing the glucose oxidase method (Pars Azmoon Co., Iran). Rats were fasted for 12 hours and anesthetized as described above. Serum samples were prepared from blood obtained from the tips of the tails by centrifugation at 5000 g for 10 minutes. The intra-assay coefficient of variation (CV) was 2.6 %. 

### Measurement of tissues' nitric oxide metabolites

The modified Griess method was employed to measure the concentrations of NOx and nitrite in all samples (Miranda et al., 2001[28]). In brief, NaOH (3.72 M) (Navarro-Gonzalvez et al., 1998[30]) and zinc sulfate (15 mg/mL) (Ghasemi et al., 2007[12]) were added to the 300 μL of homogenized tissues (100 mg of all the tissues studied in 500 μL of phosphate-buffered saline (PBS, pH 7.4) except for the brain and eAT where 200 μL of PBS was used) to prevent turbidity in the Griess reaction and to deproteinize the samples, respectively. The homogenates were centrifuged at 10,000 g for 10 minutes; the supernatants were used to measure NOx and nitrite concentrations. To determine NOx levels, vanadium trichloride (100 μL, 8 mg/mL in 1 M HCl), N-(1-naphthyl) ethylenediamine (50 μL, 0.1 % in ddH_2_O), and sulfanilamide (50 μL, 2 % in 5 % HCl), were added to the samples. For measuring nitrite levels, the following components were added to the samples: 1 M HCl (100 μL), N-(1-naphthyl) ethylenediamine (50 μL, 0.1 % in ddH_2_O), and sulfanilamide (50 μL, 2 % in 5 % HCl). NOx and nitrite concentrations were determined using a standard calibration curve ranging from 0 to 100 µM for sodium nitrate and 0 to 20 µM for sodium nitrite, respectively. The nitrite values were subtracted from the NOx concentrations to determine tissue nitrate concentrations. Protein concentration was determined by the Bradford method. The concentrations of nitrate and nitrite in the tissues are expressed as nmol/mg protein. The intra-assay CVs for nitrite and Nox were 4 % and 3 %, respectively.

### Assessment of sialin gene expression 

The TRIzol reagent (Invitrogen, USA) was used for RNA extraction from the tissue samples, and RNA quality and concentration were assessed using a NanoDrop-1000 spectrophotometer (Thermo Scientific, USA). cDNA was synthesized using a kit (SMOBiO Technology, Taiwan). The reaction mixture consisted the extracted RNA (1 μg), random hexamer (1 μL, 100 µM), dNTPS Mix (1 μL, 10 mM), RNAokTM RNase inhibitor (1μL, 20 U/μL), ExcelRTTM Reverse transcriptase (RT) (1 μL, 200 U/μL), RT buffer (4 μL), and diethyl pyrocarbonate (DEPC)-treated H_2_O (4 μL). The thermal cycling procedure consisted of a 5-minute incubation at 70 °C, followed by subsequent incubations at 25 °C for 20 minutes and at 50 °C for 50 minutes.

For cDNA amplification, a Rotor-Gene 6000 real-time PCR machine (Corbett, Life Science, Sydney, Australia) was employed along with SYBR Green PCR Master Mix 2X (Ampliqon Company, Denmark). The reaction mixture was composed of cDNA (2 μL), forward and reverse primers (2 μL), DEPC-treated H_2_O (8.5 μL), and Master Mix (12.5 μL); the total volume was 25 µL. The thermal cycling protocol began with an initial denaturation step at 95 °C for 10 minutes, followed by 40 amplification cycles under the following conditions: 94 °C for 45 seconds, 58 °C for 45 seconds, and 72 °C for 60 seconds. Duplicate runs were performed for each tissue sample, and negative control reactions were carried out using nuclease-free water in place of templates. The primer sequences for the sialin gene and the housekeeping gene, GAPDH, are listed in Table 1(Tab. 1). GAPDH is frequently used as a housekeeping gene in gene expression studies across various tissues due to its high abundance and consistent expression levels in both normal (Barber et al., 2005[4]) and T2D (Perez et al., 2017[38]) rodents.

### Statistical analysis

Data analysis was performed using GraphPad Prism software (Version 8, La Jolla, California, USA). Data are presented as mean ± SEM, except for the gene expressions of sialin. The gene expressions of sialin are presented as relative fold changes. The Dixon outlier range statistic was used to determine outliers (Horn et al., 2001[16]). In the Dixon test, if the D (absolute difference between the most extreme values)/R (range of the data) ratio exceeds 1/3, the extreme value should be deleted as an outlier (Horn et al., 2001[16]). The REST software was used to calculate relative expressions of sialin versus GAPDH (Pfaffl et al., 2002[39]). This software performed randomization tests comparing T2D and T2D+nitrate samples, avoiding data distribution assumptions and preferred over parametric tests (Pfaffl et al., 2002[39]). It also provides efficiency-corrected relative gene expression, preventing miscalculations (Rao et al., 2013[44]), and allows comparisons between groups based on reference and target genes (Pfaffl et al., 2002[39]). The Student's t-test was used to compare the nitrite and nitrate levels among the two groups. To compare the body weight and glucose levels between groups at the beginning and end of the study, a two-way mixed (between-within) analysis of variance (ANOVA) was performed. The Bonferroni post-hoc test was used for further analysis. Statistical significance was determined with two-sided P-values<0.05.

## Results

### Serum glucose and body weight 

Compared to T2D rats, the T2D+nitrate group had 18.8 % lower serum glucose levels (P=0.022) and 9.0 % lower body weight (P=0.028) at month 6 (Table 2(Tab. 2)). 

### Tissue nitrate and nitrite levels 

**Figure 2:**
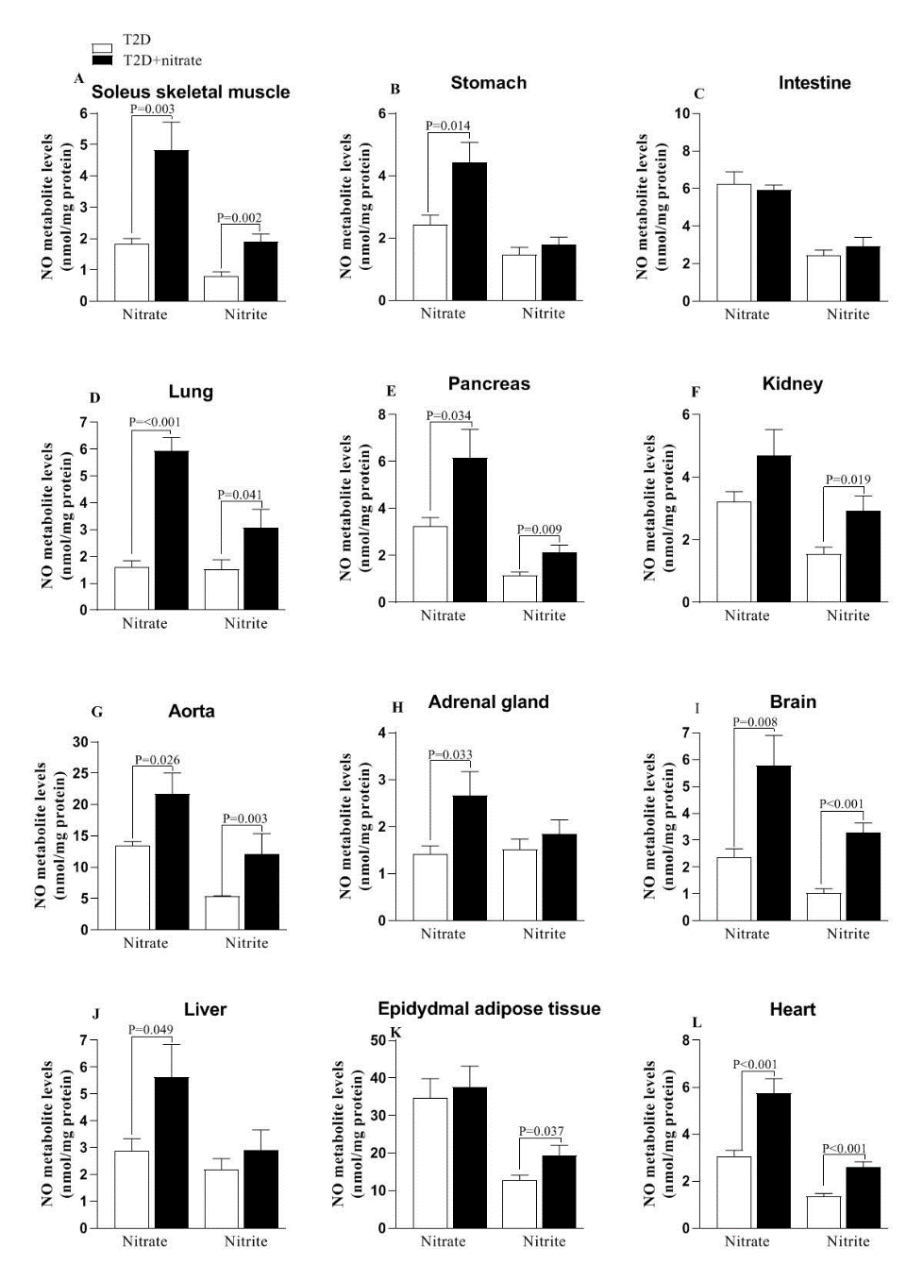
Changes in tissues' nitrate and nitrite levels in type 2 diabetes (T2D) and T2D+nitrate groups. NO, Nitric oxide

Compared to non-treated T2D rats, the nitrate-treated T2D rats had higher nitrate levels in the SM (163 %), stomach (83 %), lung (271 %), pancreas (90 %), aorta (61 %), adrenal gland (88 %), brain (145 %), liver (95 %), and heart (87 %); however, no changes were detected in the intestine, kidney, and eAT. Also, compared to the non-treated T2D rats, the T2D+nitrate group had higher nitrite levels in the SM (136 %), lung (108 %), pancreas (86 %), kidney (88 %), aorta (33 %), brain (221 %), eAT (52 %), and heart (93 %). Nitrite values in the liver, intestine, stomach, and adrenal gland were similar between T2D and T2D+nitrate rats (Figure 2(Fig. 2)).

### Tissue sialin gene expression 

**Figure 3:**
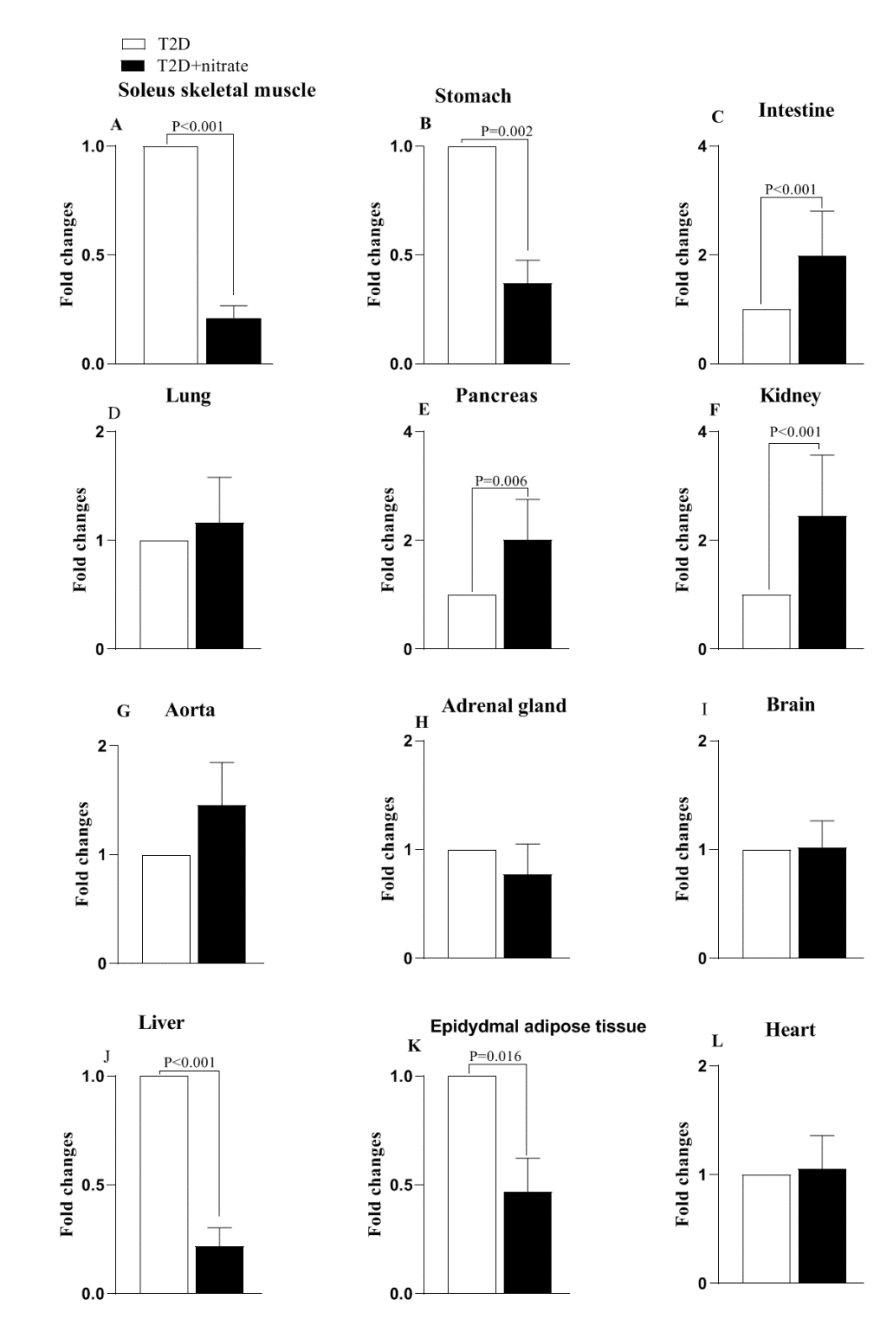
Changes in gene expression of sialin in type 2 diabetes (T2D) and T2D+nitrate groups

Sialin gene expression was significantly lower in the SM (0.21-fold), stomach (0.37-fold), liver (0.21-fold), and eAT (0.47-fold) of the nitrate-treated rats compared to the untreated rats. In comparison to the T2D group, the T2D+nitrate group demonstrated increased sialin gene expressions in the intestine (1.99-fold), pancreas (2.01-fold), and kidney (2.45-fold). No significant alterations in sialin gene expression were detected in the lung, aorta, heart, adrenal gland, and brain (Figure 3(Fig. 3)).

See also the supplementary data.

## Discussion

The results of the current study showed, for the first time, that nitrate administration to rats with T2D decreased sialin gene expression in some tissues (SM, stomach, liver, and eAT). In contrast, it increased it in others (pancreas, intestine, and kidney), with no significant alterations in the remaining studied tissues (lung, aorta, adrenal gland, brain, and heart). These findings partially confirm our hypothesis regarding the compensatory increase in sialin gene expression to counteract the reduced availability of NO in T2D.

In this study, nitrate administration decreased serum glucose and body weight in T2D rats by 18.8 % and 9.0 % at month 6, respectively. Previous reports have documented decreased fasting glucose levels and body weight in T2D rats following nitrate or nitrite administration after 8 (Gheibi et al., 2018[14]), 10 (Carlström et al., 2010[8]), 12 (Lai et al., 2016[22]; Varzandi et al., 2018[49]), and 28 (Khorasani et al., 2019[21]; Shokri et al., 2021[47]) weeks. The positive metabolic effects of nitrate have been attributed to several factors, including increased glucose uptake by skeletal muscle (Lai et al., 2016[22]), decreased insulin resistance (Nyström et al., 2012[31]), increased browning of white adipose tissue (Roberts et al., 2015[46]; Varzandi et al., 2018[49]), enhanced insulin secretion by the pancreas (Nyström et al., 2012[31]; Ghasemi et al., 2022[10]), reduced oxidative stress in the pancreatic islets (Ghasemi et al., 2023[11]), increased glucose uptake by skeletal and adipose tissue (Jiang et al., 2014[19]; Khoo et al., 2014[20]), and improved insulin sensitivity in skeletal muscle (Ohtake et al., 2015[32]). 

Based on our findings, it was observed that in all the tissues studied, except for the intestine, higher levels of nitrate (SM, stomach, lung, pancreas, aorta, adrenal gland, brain, liver, and heart) and nitrite (SM, lung, pancreas, kidney, aorta, brain, eAT, and heart) were observed in T2D rats after 6 months of nitrate administration. In line with our results, increased levels of NO metabolites have been reported in the SM (168 %) (Shokri et al., 2021[47]) and inguinal AT (95 %) (Varzandi et al., 2018[49]) of T2D rats after 3 (Varzandi et al., 2018[49]) and 6 (Shokri et al., 2021[47]) months of nitrate (100 mg/L) (Shokri et al., 2021[47]) or nitrite (50 mg/L) (Varzandi et al., 2018[48]) administration. These findings suggest that nitrate supplementation counteracts NO deficiency at the tissue level in T2D rats. Efficacy of nitrate administration for increasing tissue NO bioavailability has also been reported in normal rats, where nitrate/nitrite levels increased in gluteus muscle (1.7-fold), liver (2.4-fold), and eyes (4.2-fold) following 3 days of nitrate administration at 1 g/L (Park et al., 2023[35]). Similarly, a recent study by Piknova et al. reported that three hours after ingestion of ^15^N-nitrate (1g/L) in normal pigs, nitrate concentrations in skin, femur bone, gluteus muscle, and liver increase by ~2-2.4-fold (Piknova et al., 2024[40]). Increased NO metabolites in tissues of T2D are mediated by improving NOS-dependent NO production and activating the nitrate-nitrite-NO pathway. In support, administering nitrate to T2D rats increased mRNA and protein levels of eNOS in rats' liver, SM, and eAT (Shokri et al., 2021[47]) and decreased iNOS mRNA in their SM and eAT (Gheibi et al., 2018[14]). In addition, consuming nitrate can activate the nitrate-nitrite-NO pathway, compensating for impaired endogenous eNOS-derived NO (Lundberg et al., 2010[24]).

Our results showed that nitrate administration decreased the sialin gene expression in the SM, stomach, liver, and eAT, accompanied by increased nitrate or nitrite concentrations in these tissues. These results confirm our hypothesis that increased gene expression of sialin in some tissues (SM, stomach, liver, and eAT) is a counterregulatory response for reduced NO bioavailability in rats with T2D. In further support of this hypothesis, it has been reported that myoglobin-deficient mice have a lower reduction rate of nitrite to NO and higher (89 %) sialin expression, which acts as a counterregulatory response for increased NO bioavailability (Park et al., 2019[33]). Moreover, Park et al. have shown that sialin expression increased by 50 % in rat SM following decreased nitrate supplementation for 21 days, suggesting that elevated sialin gene expression acts as a counterregulatory in response to decreasing nitrate levels (Park et al., 2021[36]). To sum up, it seems that sialin increases the influx of nitrate into cells that boosts NO production in the states of NO deficiency. However, it has been recently reported that sialin decreases NO bioavailability in dysfunctional endothelial cells by increasing nitrate efflux (Akhtar et al., 2024[2]). Our findings in SM of diabetic rats support the hypothesis proposed by Piknova et al., which suggests that skeletal muscle, due to its large size and limited nitrate reductase activity, acts as the primary organ for nitrate storage (Piknova et al., 2022[41]). In a recent study by the same group, three criteria were suggested for organs that effectively store nitrates: a large volume, low metabolism, and well-regulated blood flow, all characteristics evident in the skeletal muscle (Piknova et al., 2024[40]). In contrast, other organs, such as the liver, are considered the main sites for nitrate consumption and reduction (Piknova et al., 2022[41]). This unique characteristic of skeletal muscle allows it to play a protective role against dietary nitrate deprivation (Piknova et al., 2022[41]). However, this compensatory mechanism cannot be generalized to all tissues in our study, and nitrate administration increased sialin gene expression in the intestine, pancreas, and kidney.

It has been suggested that nitrate positively feedbacks on its transport and vice versa in the intestine, pancreas and kidney, potentially amplifying of the nitrate-nitrite-NO pathway (Park et al., 2023[35]). It has been shown that nitrate (4 mM sodium nitrate in drinking water for 7 [47] and 180 [47] days) increases sialin protein expression, which can prevent hypoxia-induced intestinal injury in mice (Xu et al., 2024[51]) and age-related intestinal epithelial dysfunction in aged (18-month-old) mice (Wang et al., 2024[50]). Results of some *in vitro* studies also support the notion that nitrate positively feedbacks on its transport and that sialin gene expression increased in human parotid gland cells (hPGCs) following nitrate administration (0.1, 0.2, or 0.5 mmol/L for 72 hours) (Feng et al., 2021[9]). In addition, sialin gene expression decreased after adding a NO blocker to the cultures of hPGCs (Feng et al., 2021[9]). Sialin in the kidney may contribute to nitrate reabsorption (Carlström, 2021[7]), and it is speculated that increases in its expression in the kidney would be associated with elevated nitrate and nitrite levels.

The strengths of this study are that we assessed the sialin gene expression in 12 tissues of rats, providing insights into the distribution of this gene. Additionally, we used a combination of an HFD and low-dose STZ to induce conditions that mimic the pathophysiology of T2D in humans. This model effectively induces insulin resistance, relative hyperinsulinemia, stable hyperglycemia, and hypertriglyceridemia, thus closely resembling the characteristics observed in T2D patients (Reed et al., 2000[45]; Gheibi et al., 2017[15]; Ghasemi and Jeddi, 2023[13]). A limitation of our study is that we only measured sialin gene expression at the end of the research period. Additional studies at various time points could reveal nitrate administration's acute and chronic effects on sialin expression.

## Conclusion

The results of this study demonstrated that nitrate administration restores the compensatory upregulation of sialin gene expression in specific tissues (SM, stomach, liver, and eAT) of T2D rats. This compensatory mechanism is tissue-specific and nitrate administration in T2D rats elevated sialin gene expression in the intestine, kidney, and pancreas, suggesting that nitrate positively feedbacks on its transport in these tissues and vice versa.

## Declaration

### Ethical approval

The ethics committee at the Research Institute for Endocrine Sciences, associated with Shahid Beheshti University of Medical Sciences approved all experimental procedures for this study (Ethics Code: IR.SBMU.ENDOCRINE.REC.1402.075).

### Declaration of competing interest 

The authors declare that they have no competing interests.

### Data availability

The data that support the findings of this study are available from the corresponding author, upon reasonable request.

### Acknowledgment and funding information

This study was supported by a grant (Grant No. 43003490-4) from Shahid Beheshti University of Medical Sciences.

### Author contribution

Conceptualization, SJ, NY, KK, and AG; methodology, SJ, NY, VK, MZ, and AG; formal analysis, SJ, NY, VK, and AG; data curation, KK and AG; original draft preparation, SJ, NY and AG; review and editing, SJ, KK and AG; supervision, AG; project administration, AG; funding acquisition, AG. All authors have read and approved the final manuscript. 

## Supplementary Material

Supplementary data

## Figures and Tables

**Table 1:**
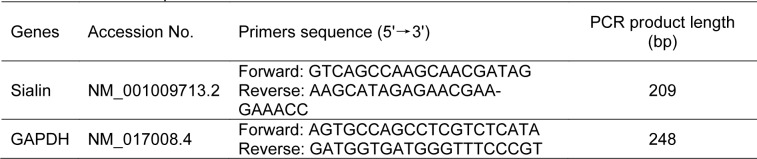
Primers sequence

**Table 2:**
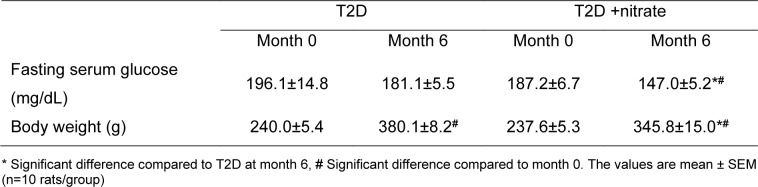
Fasting serum glucose concentrations and body weights at the beginning and end of the study in type 2 diabetes (T2D) and T2D +nitrate rats.
